# Selected transgenic murine models of human autoimmune liver diseases

**DOI:** 10.1007/s43440-021-00351-y

**Published:** 2022-01-15

**Authors:** Katarzyna Trzos, Natalia Pydyn, Jolanta Jura, Jerzy Kotlinowski

**Affiliations:** Department of General Biochemistry, Faculty of Biochemistry, Biophysics and Biotechnology, Jagiellonian University, Gronostajowa Street 7, 30-387 Krakow, Poland

**Keywords:** Autoimmune disease, Liver, Mouse models, Primary biliary cholangitis, Autoimmune hepatitis, Primary sclerosing cholangitis

## Abstract

Murine models of human diseases are of outmost importance for both studying molecular mechanisms driving their development and testing new treatment strategies. In this review, we first discuss the etiology and risk factors for autoimmune liver disease, including primary biliary cholangitis, autoimmune hepatitis and primary sclerosing cholangitis. Second, we highlight important features of murine transgenic models that make them useful for basic scientists, drug developers and clinical researchers. Next, a brief description of each disease is followed by the characterization of selected animal models.

## Autoimmune diseases—introduction

The basis of autoimmune diseases is the loss of the organism’s ability to distinguish self-antigens from nonself-antigens. When it happens, the immune system is stimulated and during adaptive immune response, organ-specific T and B cells are produced and triggered to react with selected self-antigens. As a result, immune tolerance of a mentioned organ has been broken. There are more than 80 types of autoimmune diseases affecting almost any possible organ or tissue. Approximately 3–5% of the population is affected by autoimmune diseases. Among autoimmune diseases of the digestive system, celiac disease and Crohn’s disease are the most common. Rheumatoid arthritis (incidence 9–45 per 100,000 person-years globally), Graves’ disease (incidence 21–120 per 100,000 person-years globally), and type 1 diabetes (incidence 1–20 per 100,000 person-years globally) are also very common [1]. The prevalence of autoimmunity is constantly rising, and the reason for this remains unknown [1–3]. The liver is not an exception, and the three major categories of autoimmune liver diseases (ALDs) are primary biliary cholangitis (PBC), autoimmune hepatitis (AIH) and primary sclerosing cholangitis (PSC). Although each of these diseases has well-defined clinical, morphologic, and serologic profiles (Table 1), atypical forms and overlap syndromes are common, making both diagnosis and treatment challenging [4].

**Table 1:** Comparison of autoimmune liver diseases: primary biliary cholangitis, autoimmune hepatitis and primary sclerosing cholangitis

Feature	Disease		
	PBC	AIH	PSC
Female: male ratio	9:1	4:1 (type I), 10:1 (type II)	1:2
Autoantibodies	AMA, ANA	ANA, ASMA, ASLA/LP, ALKM1, ALKM3, ALC1	AMA, pANCA
Blood test abnormalities	AST and ALT elevated	ALT elevated	ALP and ALT elevated
Hyperglobulinemia	Yes	Yes	Yes
Liver histology	Destruction of the intrahepatic bile ducts, fibrosis, cholestasis, cirrhosis	Destruction of the parenchyma, chronic fibrosis, cirrhosis	Inflammation of the bile ducts, fibrosis, cirrhosis
HLA association	HLA-DRB1*08	HLA-A*03, HLA-B*08, HLA-DRB1*03, HLA-DRB1*04	HLA-DRA*01:DRB3
Treatment	Bile acids (UDCA, OCA), liver transplantation	Immunosuppression (prednisone, azathioprine), liver transplantation	Immunosuppression (corticosteroids, azathioprine), liver transplantation

Abbreviations: *AMA* anti-mitochondrial antibodies, *ANA* anti-nuclear antibodies, *ASMA* anti-smooth muscle autoantibodies, *ASLA/LP* autoantibodies specific to a soluble liver or pancreas antigen, *ALKM1* antibodies against type 1 hepatorenal microsomes, *ALKM3* antibodies against type 3 hepatorenal microsomes, *ALC1* autoantibodies against type 1 liver cytosol, *pANCA* atypical perinuclear anti-neutrophil cytoplasmic antibodies, *ALP* alkaline phosphatase, *ALT* alanine aminotransferase, *AST* aspartate aminotransferase, *UDCA* ursodeoxycholic acid, *OCA* obeticholic acid

Current biomarkers cannot accurately identify individuals that will develop autoimmune disorders, but there are risk factors that increase the possibility of getting sick. Viral infections, environmental factors, family history, ethnic background and genetic factors associated with human leukocyte antigens (HLAs) are the most important risk factors [5, 6]. The co-occurrence of risk factors, different exposures to them (i.e., dose, time), and personal susceptibility to disease produce many different autoimmune disease courses. In addition, it is known that many types of autoimmune diseases occur much more often in women than in men [7]. Although PBC, AIH and PSC are classified as autoimmune diseases, factors leading to their development are not fully understood. Nevertheless, several risk factors associated with ALDs have been identified that help to predict disease outcome.

## Etiology and risk factors

In the case of AIH and PBC, it is believed that contact with hepatitis viruses (types A, B and C), Epstein–Barr virus and herpes simplex virus can lead to the development of the disease [5]. The occurrence of molecular mimicry, i.e., structural similarities between the antigens of the host and the pathogen, contributes to the breakdown of immune tolerance in the liver. Contact with hepatitis C virus (HCV) may increase the likelihood of developing an autoimmune response in a patient, because antibodies binding to selected HCV proteins (generated in the course of infection), may cross-react with the autoantigen *CYP2D6*. As a result, immune tolerance is broken, for example, during AIH type II [5]. Since *CYP2D6* was proven to have homologous regions with Herpes virus and Cytomegalovirus genome, infection with such viruses could trigger loss of tolerance and autoimmunity [6]. Interestingly, CYP2D6*4 and CYP2D6*5 are loss of function alleles of *CYP2D6*, which are expressed by 10–33.4% and 1–6% of Europeans, respectively [8]. Lack of functional *CYP2D6* alleles may protects from autoimmune diseases, but more research is needed to support this hypothesis.

**Table 2:** Characterization of murine genetic models of PBC

Feature	Model				
	dnTGF-βRII	IL-2Rα^−/−^	Ae2a,b^−/−^	ARE-Del^−/−^	Mcpip1^fl/fl^Alb^Cre^
Genetic mutation	Overexpression of dominant-negative form of TGF-β receptor type II under the control of the CD4 promoter	Deletion of IL-2Rα	Disruption of the 3 major *Ae2* variant isoforms (*Ae2a, Ae2b1*, and *Ae2b2*)	Deletion of the adenylate–uridylate-rich element (ARE) of the *IFNG* 3′-untranslated region	Deletion of *Zc3h12a* in liver epithelial cells
Blood					
Autoantibodies	AMA	ANA, AMA	AMA	AMA	ANA, AMA
Blood test abnormalities	Elevated IL-12, IL-6, TNF-α and IFN-γ	Elevated IL-2, IL-12, IL-6, TNF-α and IFN-γ	Elevated ALP and ALT	Elevated TBA, ALT and AST	Elevated TBA and ALP
Hyperglobulinemia	n.a	Elevated IgG and IgA	Elevated IgG and IgM	n.a	Elevated IgG and IgM
Liver					
Fibrosis	n.a	n.a	Yes	Yes	Yes
Bile duct injury	Yes	Yes	Yes	Yes	Yes
Inflammation	Yes, mainly CD4, CD8, and CD19 cells, monocytes, macrophages	Yes, mainly CD4 and CD8 cells, monocytes	Yes, mainly CD4, and CD8, B cells, eosinophils and macrophages	Yes, mainly lymphoid cells	Yes, mainly CD4 and CD8 cells, macrophages and neutrophils
Other				Granuloma formation	Granuloma formation

Abbreviations: *AMA* anti-mitochondrial antibodies, *ANA* anti-nuclear antibodies, *ALP* alkaline phosphatase, *ALT* alanine aminotransferase, *AST* aspartate aminotransferase, *TBA* total bile acids, n.a. not analysed

**Table 3:** Characterization of murine genetic models of AIH

Feature	Model		
	NTxPD-1–/–	TAM–/–	Aire–/–
Genetic mutation	PD-1 knock-out (+ thymectomy)	Tyro3, Axl and Mer tyrosine kinase knock-out	Autoimmune regulator Aire knock-out
Blood			
Autoantibodies	ANA	ANA, ASMA	Polyclonal humoral immune responses against multiple liver (auto)antigens
Blood test abnormalities	AST, ALT and total bilirubin elevated	AST and ALT elevated	TNF-α, IL-2, IL-9 elevated
Hyperglobulinemia	No	Yes	No
Liver			
Bile duct injury	No	n.a.	n.a.
Inflammation	Severe mononuclear cell infiltration in the portal area and parenchyma without bile duct destruction	Severe portal inflammation, infiltration into parenchymal regions, macrophages and CD4 + and CD8 + T cells (percentage of CD4 + cells is approximately threefold more than CD8 + cells) and relatively few B cells infiltrating the liver parenchymal regions	Intrahepatic infiltrates dominated by B cells (40–50%) and CD4 + T cells with very few CD8 + cells; NK cells also present
Other	Massive degeneration of hepatocytes that exhibit vacuolar and vesicular cytoplasmic changes; most of the remaining hepatocytes show reduction of cytoplasm and nuclear condensation		

Abbreviations: *ANA* anti-nuclear antibodies, *ASMA* anti-smooth muscle autoantibodies, *ALT* alanine aminotransferase, *AST* aspartate aminotransferase, n.a. not analysed

**Table 4:** Characterization of murine genetic models of PSC

Feature	Model		
	Mdr2 –/–	Cftr –/–	fch/fch
Genetic mutation	Knock-out of the multidrug resistance gene (*Mdr2*, alias)	Knock-out of cystic fibrosis transmembrane conductance regulator (Cftr)	Homozygous point mutation in the ferrochelatase gene
Blood			
Autoantibodies	n.a.	ANA, ASMA	n.a.
Blood test abnormalities	TNF-α and TGF-β1 elevated	AST and ALT elevated	AST, ALT, bile salts and conjugated bilirubin elevated
Liver			
Fibrosis	Yes	Yes	Yes - referes to "fibrosis; Ductular reaction, mallory bodies, hepatocellular damage and loss, proliferation of atypical reactive ductules, large cell dysplasia in parenchyma, small cell dysplasia in the periportal region
Bile duct injury	Separation of the peribiliary plexus from the biliary epithelium, leading to atrophy and death of bile duct epithelial cells, cholangitis	Focal biliary cirrhosis, advanced lobular cirrhosis	
Inflammation	Yes, regurgitation of toxic bile from leaky bile ducts into the periportal tissue, leading to induction of inflammation	Yes (within and surrounding focal biliary canaliculi and bile ducts)	n.a.

Abbreviations: *ANA* anti-nuclear antibodies, *ASMA* anti-smooth muscle autoantibodies, *ALT* alanine aminotransferase, *AST* aspartate aminotransferase,  n.a not analysed

Exposure to certain environmental factors, such as sunlight, pollution, tobacco smoke, toxins, hair dyes, nail polishes, drugs or pathogens, can also lead to the development of an autoimmune response [9–12]. Environmental influence is likely to play a significant role in driving PBC, interacting with immunogenetic and epigenetic risks [13, 14]. Recently, an interesting study screened urban landfill and control soil samples from a region with high PBC incidence for potential chemical substance(s) that may cause PBC. Probert and coworkers identified for the first time a xenobiotic in the environment directly correlating with PBC development. The chemical was identified as an ionic liquid [3-methyl-1-octyl-1H-imidazol-3-ium]^+^, and the toxic effects were recapitulated with the pure chemical [15]. AIH may also develop due to the overuse of certain drugs (atorvastatin, diclofenac, isoniazid, methyldopa, minocycline, nitrofurantoin, and propylthiouracil) as a consequence of drug-induced liver injury [5].

As shown recently, the gut microbiota composition plays a critical role in influencing predisposition to various chronic liver disorders [16, 17]. Recent progress in understanding the composition and function of the human microbiota has revealed its important role in immune homeostasis [18]. This fact is not surprising, because the liver receives most of its blood supply from the intestine and is exposed to microbes and microbial products from the gut. One of the mechanisms leading to autoimmune disease development starts with leakage through the colon membrane, which in turn causes lipopolysaccharides (LPS) to enter the liver and stimulate Toll-like receptors. As a result of these events, the liver immune response is induced, resulting in leukocyte activation and infiltration into the liver parenchyma [19].

Importantly, Tang and coworkers reported substantial differences in the composition and function of the gut microbiome between treatment-naive PBC subjects and healthy controls. A significant reduction in individual microbial diversity was noted in PBC subjects. Such patients were also defined by decreased abundances of four genera (*Bacteroidetes, Sutterella, Oscillospira, Faecalibacterium)* and increased abundances of eight genera (*Haemophilus, Veillonella, Clostridium, Lactobacillus, Streptococcus, Pseudomonas, Klebsiella*, *Enterobacteriaceae*) [20]. There have also been studies confirming the occurrence of dysbiosis in the course of AIH and PSC progression. Recently, Wei and coworkers demonstrated that the composition of the intestinal microflora differs in patients suffering from AIH in comparison to that in healthy controls. They detected a decrease in the content of obligate anaerobes, as well as an increase in the content of potentially pathogenic strains, such *as Veillonella dispar* [21]*.* Notably, anaerobic bacteria are particularly important to the human digestive tract due to their ability to produce short-chain fatty acids, which have anti-inflammatory properties. Disturbance in the composition of the microbiome may cause excessive proliferation of facultative aerobic strains, leading to the development of diseases [21]. In the course of PSC, dysbiosis has also been observed and related to the increased contents of *Enterococcus*, *Fusobacterium*, *Streptococcus*, and *Lactobacillus* species and, at the same time, decreased microbial diversity [17].

In addition, it has been shown by many studies that family history and genetic predisposition are important risk factors for the development of ALDs. For example, in monozygotic twins, the concordance rate of PBC is very high—it is estimated at 63%, and for the first-degree relatives of PBC patients, it is 4% [10, 11]. It has also been demonstrated that sisters of female PBC patients had a 14-fold higher risk of developing this disease than the general population [22]. In addition, 1–2% of patients’ children develop PBC [23]. For AIH, a higher incidence among relatives occurs, but no significant increase in the risk of this disease has been documented. This is probably because it is a multigene disease, which results in a lower chance of heredity [24]. In line with these observations, in AIH monozygotic twins, the concordance rates were only approximately 4–5% (1 of 22 pairs of twins). There is also a link between the risk of AIH development and the DRB1 region of the human leukocyte antigen (HLA) complex [24]. Likewise, a similar link exists in the case of PBC and applies to the DR7 and DR8 regions [11]. Moreover, for PSC, genetic background is a major risk factor. First-degree relatives of patients suffering from PSC have an approximately ten times higher risk of developing PSC [25]. Genetic risk factors identified thus far for this disease are mainly related to HLA on chromosome 6. Karlsen et al. also identified candidate risk genes outside HLA on chromosome 6, such as *MMEL1*, *TNFRSF14*, *IL2RA*, *TCF4*, and *CD226* [25]

## Murine models of human diseases

ALD models can be divided into three main groups: knockout murine models, models of chemically induced disease and infection-triggered disease models. Unfortunately, none of the currently existing animal models can fulfill all traits of human ALDs. Given the complex etiology and progression of ALDs, different animal models should be used to shed new light on these diseases. Since no single model can be used to fully address key features of PBC, AIH or PSC, there is still a need for the development of new models or orthogonal approaches.

In this review, we discuss murine models in which the development of ALD is induced by specific genetic manipulation. An ideal animal model should recapitulate the main traits of human PSC: (i) fibrosis of both intra- and extra-hepatic bile ducts with a characteristic ‘onion skin’ pattern of collagen deposition; (ii) leukocyte infiltration; (iii) association with inflammatory bowel disease; and (iv) predisposition for cholangiocarcinoma development. In terms of PBC, features that should be represented by the experimental model include (i) involvement of small interlobular bile duct pathology; (ii) peribiliary leukocyte infiltration; (iii) granuloma formation; (iv) female predominance; and (v) presence of antimitochondrial antibodies (AMAs) [26]. Ideally, an experimental model of AIH would be characterized by (i) leukocyte infiltration into the liver parenchyma; (ii) fibrosis; (iii) the presence of autoantibodies; and (iv) hypergammaglobulinemia [27].

## Primary biliary cholangitis

PBC is a chronic liver disease that results from slow, progressive destruction of the intrahepatic bile ducts. PBC progression leads to the development of fibrosis, cholestasis and, in some people, liver cirrhosis [28]. Depending on the stage of the disease, histological examination may reveal portal inflammation, bile duct hyperplasia and obstruction, hepatitis, or cirrhosis [11]. Clinically, four stages of PBC have been distinguished: (i) portal inflammation with or without florid bile duct lesions, (ii) an increase in the size of periportal lesions with interface hepatitis, (iii) distortion of hepatic architecture with numerous fibrous septa, and (iv) cirrhosis [29].

Currently, we know that one of the key features of PBC is the presence of autoantibodies against nuclear and mitochondrial antigens (Table 1). Consequently, scientists and physicians claim that PBC development arises from loss of tolerance to proteins that are normally located within cells, such as the E2 component of the mitochondrial pyruvate dehydrogenase complex (PDC-E2), which is present in the inner mitochondrial membrane [10, 11]. In PBC patients, autoantibodies against glycoprotein 210 (gp210, mitochondrial protein) and Sp100 nuclear antigen are also often detected. Mechanistically, during apoptosis of cholangiocytes, immunologically intact PDC-E2 is present within apoptotic blebs. This leads to immune system attack on cholangiocytes, which then results in bile duct destruction, followed by enhanced leukocyte infiltration and liver fibrosis, which can ultimately lead to cirrhosis and liver failure [10, 30].

There is only one FDA-approved drug for PBC treatment, ursodeoxycholic acid (UDCA), which is a naturally occurring hydrophilic bile acid [31]. UDCA treatment improves liver functional tests slows disease progression and can partially reverse dysbiosis [20]. Nevertheless, UDCA does not completely cure the disease, and approximately 40% of patients do not respond to treatment with UDCA [32]. The second therapy line, recommended by the European Association to Study Liver (EASL), implements the usage of obeticholic acid (OCA) or fibric acid derivatives/budesonide in combination with UDCA; however, none of these drugs is able to cure PBC [10–12]. The only definite treatment for PBC is liver transplant, and in Europe, it is the third most common reason for performing this procedure. However, even after transplantation, AMA levels are positive in most patients, and the recurrence rate at 15 years is 40%.

## Transgenic murine models of  primary biliary cholangitis

One of the first PBC genetic models described was dnTGF-βRII mice with overexpression of the dominant negative form of TGF-*β* receptor type II under the control of the CD4 promoter, which results in almost complete abrogation of TGF-*β* signaling in CD4 + T cells. As a consequence, dnTGF-*β*RII mice develop PBC symptoms, such as the presence of AMAs in serum and bile duct injury. Moreover, these mice are characterized by infiltration of monocytes/macrophages and lymphoid cells (CD4^+^, CD8^+^ and CD19^+^ lymphocytes) in portal tracts of the liver [33] (Table 2).

Another example of a PBC model is IL-2R*α* − / − mice, characterized by the blockage of IL-2 signaling. Above all, this deletion results in disruption of Treg cell differentiation, which is believed to protect against autoimmunity. All IL-2R*α* − / − mice are AMA positive (100% incidence), and in 80% of them, ANAs can be detected. In addition, the levels of proinflammatory cytokines, such as IL-2, IL-12, IL-6, TNF-*α* and IFN-*γ*, are elevated in the serum of these mice. Liver symptoms of PBC in IL-2R*α* − / − mice include damage to bile ducts, particularly interlobular bile duct destruction, and infiltration of lymphoid (CD4^+^ and CD8^+^ lymphocytes) and monocytic cells in portal tracts [34]

Anion exchanger 2 (Ae2) is a protein involved in biliary bicarbonate excretion and, therefore, bile flow. By disruption of the three major Ae2 variant isoforms (Ae2a, Ae2b1, and Ae2b2), Salas and coworkers developed a murine model of human PBC. The serum of Ae2a,b–/– mice is characterized by increased levels of IgG and IgM antibodies, an increase in alkaline phosphatase activity, and the presence of AMAs against PDC-E2. Furthermore, PBC features, such as bile duct damage, cholestasis, fibrosis and inflammation (infiltration of CD4 + cells, CD8 + cells, B cells, eosinophils and macrophages) are observed in the livers of these mice [35].

To the best of our knowledge, there is only one murine model—IFNγ-ARE-Del mice—that mimics the female predominance of PBC that is present in humans [36]. IFN*γ*-ARE-Del mice are characterized by dysregulation of IFN-*γ* signaling caused by the deletion of the adenylate–uridylate-rich element (ARE) of the *IFNG* 3′-untranslated region. This modification results in the constitutive and persistent production of IFN-*γ*. The levels of AMAs and bile acids, as well as the activity of alanine aminotransferase (ALT) and aspartate aminotransferase (AST), are elevated in the sera of ARE-Del–/– mice. Moreover, hepatic PBC manifestations such as bile duct destruction, granuloma formation, inflammation and fibrosis are more severe in female mice than in male mice and, therefore, reflect the sex bias of PBC observed in human patients [36].

The newest mouse model resembling human PBC was described by us recently. Mcpip1^fl/fl^Alb^Cre^ mice are characterized by deletion of the *Zc3h12a* gene (encoding Mcpip1 protein) in liver epithelial cells. Serum analysis revealed increased activity of alkaline phosphatase and elevated concentrations of total bile acids in 6-week-old Mcpip1^fl/fl^Alb^Cre^ mice. In addition, levels of total IgG, IgM and AMA against PDC-E2 were also elevated. Bile duct pathology in Mcpip1^fl/fl^Alb^Cre^ mice included hyperplasia of the intrahepatic bile ducts, bile duct epithelium disruption and fibrosis, resulting in lumen obstruction and bile duct destruction. The livers of these mice were characterized by granuloma formation and parenchymal inflammation with concomitant infiltration of lymphocytes (CD4^+^ and CD8^+^), macrophages and neutrophils [37].

## Autoimmune hepatitis

AIH is a chronic, immune-mediated disease with progressive inflammation and necrosis of the liver. Unlike PSC and PBC, this disease causes pathologies in the liver parenchyma and not in bile ducts. Successive destruction of hepatocytes over time leads to liver fibrosis. The main symptoms of this disease are the presence of autoantibodies, hypergammaglobulinemia, and elevated serum transaminase activity. The presence of specific autoantibodies distinguishes type I and type II AIH [5, 27].

A specific feature of type I AIH is the presence of antinuclear autoantibodies (ANAs), anti-smooth muscle autoantibodies (ASMAs) and autoantibodies specific to a soluble liver or pancreas antigen (ASLA/LP). Histological evaluation of the liver shows destruction of the parenchyma and development of chronic fibrosis [4]. Type I AIH accounts for 90% of all AIH cases. It is most common in young women (female-to-male ratio: 4:1) [38]. In type II AIH patients, antibodies against type 1 and 3 hepatorenal microsomes (ALKM1, ALKM3) and/or against type 1 liver cytosol (ALC1) are present. In addition, in this type of AIH, pathological changes in hepatocytes are present, which can be diagnosed by histological examination [39]. Type II AIH is less common than type I AIH and is most often diagnosed in children and adolescents (female-to-male ratio: 10:1) [38].

The course of treatment for AIH is strictly dependent on the stage of the disease at the time of diagnosis. There are several treatments for AIH that are recommended by the EASL and American Association for the Study of Liver Diseases (AASLD). They consist of the administration of prednisone and, after a certain period, the administration of prednisone and azathioprine. Both drugs have immunosuppressive properties [40]. Correct diagnosis and implementation of appropriate treatment is able to guarantee that patients with type I AIH have a high quality of life. Regarding type II AIH, treatment can often be ineffective, and relapses occur [40].

## Transgenic murine models of autoimmune hepatitis

Currently, there are several transgenic murine models that develop a disease characterized by symptoms very similar to those of AIH occurring in humans [27]. The first described transgenic mouse model that develops AIH-like disease is the NTxPD-1–/– model. The model is based on the knockout of the gene encoding programmed death receptor 1 (*PD-1*), which is responsible, inter alia, for the negative regulation of autoreactive and pathogenic T lymphocytes. Newborn mice are thymectomized to significantly reduce the number of regulatory T cells. As a result, NTxPD-1–/– mice are characterized by acute lymphopenia of T lymphocytes, especially in the periphery. NTxPD-1–/– mice produce ANAs, the parenchyma is infiltrated by CD4 + and CD8 + T cells, lobular necrosis is present, and fatal hepatitis occurs around the third week of life [41] (Table 3).

Another model was created with mice devoid of genes responsible for the expression of Tyro3, Axl and Mer tyrosine kinases (the so-called TAM–/– model). All of these proteins are responsible for the negative regulation of the TLR3 intracellular signaling pathway. Thus, in TAM–/– mice, the TLR3 pathway is overactivated, resulting in the subsequent release of proinflammatory cytokines, such as IFN-*α* and TNF-*α*, which in turn enhance the expression of CXCL9 and CXCL10. These two chemokines are essential for the induction of T-cell infiltration into the liver. Consequently, hepatitis develops in TAM–/– mice with concomitant production of ANAs and ASMAs, elevated serum transaminase activity, portal inflammation and necrosis [42].

Another mouse transgenic model that is used for research on AIH is a knockout strain deficient in the autoimmune regulator Aire (Aire–/–). Aire is a transcriptional regulator that can influence the expression of certain autoantigens, resulting in the depletion of autoreactive T cells and stimulating the production of regulatory T cells [43]. In Aire–/– mice, autoimmune reactions develop against selected organs, including the liver. These mice exhibit symptoms characteristic of AIH, such as lymphocytic infiltration, elevated serum transaminase activity, and the presence of autoantibodies. The symptoms of AIH have been shown to develop more intensely in Aire–/– BALB/c mice than, for example, Aire-deficient mice with a C57BL/6 genetic background [44].

## Primary sclerosing cholangitis

PSC is a chronic autoimmune disease in which there is progressive inflammation of the bile ducts, resulting in fibrosis. Autoantibodies and hypergammaglobulinemia are also present. Advanced-stage PSC is characterized by liver cirrhosis, which is an indication for transplantation. In the course of the disease, T cells actively destroy the cells of the biliary epithelium. An inflammatory infiltrate is formed, mainly consisting of CD4 + T cells, neutrophils, and macrophages [4, 19].

Most patients remain asymptomatic at the moment of diagnosis. Unlike AIH and PBC, a diagnosis of PSC cannot be made based on the presence of specific autoantibodies. While only 20% of patients with PSC have AMAs, the majority have atypical perinuclear anti-neutrophil cytoplasmic antibodies (pANCAs), and these are the only autoantibodies that can be considered diagnostic features of PSC. However, there is no correlation between p-ANCA and clinical parameters or the clinical spectrum of the disease [45]. Biochemical tests can also be useful in diagnosis, and in most cases, increased activity of serum ALP and AST support PSC diagnosis. However, unequivocal diagnosis of PSC is based on magnetic resonance cholangiopancreatography; visualization of the characteristic multifocal structures and dilatations within the gallbladder is indicative of PSC [4, 19]. Interestingly, a higher incidence of PSC in 30–40-year-old men (female-to-male ratio 1:2) than in women has been previously reported [4]. However, it is believed that this disease is as common in males as it is in females but is much milder in females and is, therefore, less frequently diagnosed [25].

Treatment usually begins with corticosteroids and continues with azathioprine. Sometimes both are administered at the same time[4]. There are some possibilities for future PSC management based on, for example, the role of farnesoid X receptor agonists, bile salt transporter protein inhibitors and the use of monoclonal antibodies, antibiotics or UDCA. Unfortunately, for now, only liver transplantation ensures full recovery from PSC [19].

## Transgenic murine models of primary sclerosing cholangitis

One of the murine transgenic models designed for the study of PSC is a strain with a knockout of multidrug resistance gene 2 (*Mdr2*, *alias Abcb4*). Mdr2–/– mice develop PSC-like disease in a complex process that begins with the leakage of bile acids from the bile ducts into portal tracts, subsequent inflammation, fibrosis, and finally apoptosis of biliary epithelial cells. There is also infiltration of lymphocytes (neutrophils and CD4 + and CD8 + T lymphocytes) into the liver and overexpression of the TNF-*α*, IL-1*β*, IL-6, and TGF-*β*1 cytokines. Mdr2–/– mice fully develop periductal fibrosis by 4 weeks of age [46] (Table 4).

Another model useful in PSC studies is Cftr–/– mice, which develop cystic fibrosis-like disease. Cystic fibrosis transmembrane conductance regulator (Cftr) is a membrane protein with the properties of an ion channel protein that is able to conduct chloride ions across epithelial cell membranes [47]. Knockout of Cftr leads to infiltration of inflammatory cells into the bile ducts and steatosis in 2-month-old mice. After 3 months of age, Cftr–/– animals show pathological changes in the liver, including cholangitis and periportal and bridging fibrosis [48].

Mice with a BALB/c genetic background carrying a point mutation in the ferrochelatase gene are another example of a transgenic model strain developing PSC-like disease. Mutation of fch/fch mice leads to deficiency of ferrochelatase activity and deposition of toxic protoporphyrin in the liver parenchyma and bile ducts and lobular and portal macrophages in the lumen of the small bile ducts. Damage to biliary epithelial cells and Mallory bodies are also present. Incomplete obstruction of the intrahepatic biliary tree present in fch/fch mice results in chronic cholestatic conditions. These mice also have elevated serum activity of ALT and AST as well as bile salts and conjugated bilirubin level. High levels of hepatocellular damage and loss occur in mice up to 20 weeks of age and then gradually decrease in intensity [49].

## Conclusions and future directions

ALDs are a group of pathologies of complex etiology, relatively low incidence and moderate therapeutic options. Diseases such as AIH, PBC or PSC might be silent for many years, and their diagnosis is additionally hampered by a lack of specific clinical symptoms. From clinical studies, we have learned more about their progression and treatment in recent years. However, there are still many unanswered questions, such as how to predict their onset and how to prevent ALD in a susceptible individual. Hopefully, by utilizing animal and in particular murine models of ALDs, we can learn more about the molecular mechanisms responsible for the initiation and development of ALDs. Such models are also of utmost importance for testing new drugs and for drug repurposing. We think that another important challenge to overcome in the near future is the improvement of early ALD diagnosis. For example, by utilizing new and efficient biomarkers (i.e., macrovesicles from serum), physicians may be equipped with tools for early and clear-cut diagnosis. As a result, patients will be offered timelier effective treatment options, resulting in improved disease outcomes. In such scenarios, liver failure and liver transplantation would no longer be needed.

Despite development of numerous animal models of human diseases, there are important differences between humans and mice in physiology, genetics, anatomy etc. which negatively impact transferability of findings based on animal models [50]. Indeed, as we see in clinical trials, many findings have not been successfully translated to humans, e.g., NXY-0591 or calcium channel blockers, which were considered neuroprotective in mice, were not beneficial for people with stroke [51, 52]. In addition, orthologous genes not always share similar functions, e.g., it was found that 22.5% of 120 murine orthologs of essential human genes were not indispensable in mice [53]. In addition, only about 30% of transcription factor binding sites, are identical between murine and human liver cells [54].

Moreover, microbiome of laboratory mice can impact results of experiments. Despite huge similarities (90% and 89% in phyla and genera) between human and murine microbiome, quite few differences can be depicted. For instance, these two species differ in *Firmicutes* to *Bacteroidetes* ratio, and also composition of these phyla diverge between human and mice. In addition, there are some genera which are exclusively present in humans (e.g., *Faecalibacterium*) or in rodents (e.g., *Mucispirillum*). These differences can influence the composition of white blood cells of peripheral immune system. For example it was shown that in contrary to humans, there are more lymphocytes than neutrophils in murine blood and mice are less susceptible to infections after surgery, than humans [55]. In addition, as a consequence, microbial metabolite production differ between humans and mice, e.g., murine microbiota produces more lactate, and human microbiota produces more acetate and propionate [56]. Furthermore, experiments under SPF conditions may not be relevant. Recent findings on mice exposed to natural environmental pathogens revealed higher frequency of CD8 + and CD4 + T cells, innate lymphoid cells and myeloid populations in tissues and elevation of serum immunoglobulins in comparison to SPF-housed mice, which made them more similar to adult humans [57].

Despite limitations, animal models are still the most suitable for biomedical research. However, recent developments including organ-on-chip or organoid technologies are giving hope for more accurate recapitulation of events occurring during human diseases.

## Abbreviations

ALDs: Autoimmune liver diseases
AIH: Autoimmune hepatitis
ALP: Alkaline phosphatase
ALT: Alanine aminotransferase
AST: Aspartate aminotransferase
PBC: Primary biliary cholangitis
PSC: Primary sclerosing cholangitis
ANA: Anti-nuclear antibodies
AMA: Anti-mitochondrial antibodies
HLA: Human leukocyte antigen
UDCA: Ursodeoxycholic acid
OCA: Obeticholic acid
